# The impact of the COVID-19 pandemic on maternal and perinatal health: a scoping review

**DOI:** 10.1186/s12978-021-01070-6

**Published:** 2021-01-18

**Authors:** Bethany Kotlar, Emily Michelle Gerson, Sophia Petrillo, Ana Langer, Henning Tiemeier

**Affiliations:** 1 Harvard T.H. Chan School of Public Health, Boston, MA USA; 2 George Washington University, Washington, DC USA; 3 Brown University, Providence, Rhode Island USA; 4 Department of Social and Behavioral Science, Harvard T.H. Chan School of Public Health, 677 Huntington Ave., Boston, MA 02115 USA

**Keywords:** COVID-19, SARS-CoV-2, Maternal health, Newborn health, Maternal-child transmission, Mental health, Gender equity

## Abstract

**Introduction:**

The Covid-19 pandemic affects maternal health both directly and indirectly, and direct and indirect effects are intertwined. To provide a comprehensive overview on this broad topic in a rapid format behooving an emergent pandemic we conducted a scoping review.

**Methods:**

A scoping review was conducted to compile evidence on direct and indirect impacts of the pandemic on maternal health and provide an overview of the most significant outcomes thus far. Working papers and news articles were considered appropriate evidence along with peer-reviewed publications in order to capture rapidly evolving updates. Literature in English published from January 1st to September 11 2020 was included if it pertained to the direct or indirect effects of the COVID-19 pandemic on the physical, mental, economic, or social health and wellbeing of pregnant people. Narrative descriptions were written about subject areas for which the authors found the most evidence.

**Results:**

The search yielded 396 publications, of which 95 were included. Pregnant individuals were found to be at a heightened risk of more severe symptoms than people who are not pregnant. Intrauterine, vertical, and breastmilk transmission were unlikely. Labor, delivery, and breastfeeding guidelines for COVID-19 positive patients varied. Severe increases in maternal mental health issues, such as clinically relevant anxiety and depression, were reported. Domestic violence appeared to spike. Prenatal care visits decreased, healthcare infrastructure was strained, and potentially harmful policies implemented with little evidence. Women were more likely to lose their income due to the pandemic than men, and working mothers struggled with increased childcare demands.

**Conclusion:**

Pregnant women and mothers were not found to be at higher risk for COVID-19 infection than people who are not pregnant, however pregnant people with symptomatic COVID-19 may experience more adverse outcomes compared to non-pregnant people and seem to face disproportionate adverse socio-economic consequences. High income and low- and middle-income countries alike faced significant struggles. Further resources should be directed towards quality epidemiological studies.

**Plain English summary:**

The Covid-19 pandemic impacts reproductive and perinatal health both directly through infection itself but also indirectly as a consequence of changes in health care, social policy, or social and economic circumstances. The direct and indirect consequences of COVID-19 on maternal health are intertwined. To provide a comprehensive overview on this broad topic we conducted a scoping review. Pregnant women who have symptomatic COVID-19 may experience more severe outcomes than people who are not pregnant. Intrauterine and breastmilk transmission, and the passage of the virus from mother to baby during delivery are unlikely. The guidelines for labor, delivery, and breastfeeding for COVID-19 positive patients vary, and this variability could create uncertainty and unnecessary harm. Prenatal care visits decreased, healthcare infrastructure was strained, and potentially harmful policies are implemented with little evidence in high and low/middle income countries. The social and economic impact of COVID-19 on maternal health is marked. A high frequency of maternal mental health problems, such as clinically relevant anxiety and depression, during the epidemic are reported in many countries. This likely reflects an increase in problems, but studies demonstrating a true change are lacking. Domestic violence appeared to spike. Women were more vulnerable to losing their income due to the pandemic than men, and working mothers struggled with increased childcare demands. We make several recommendations: more resources should be directed to epidemiological studies, health and social services for pregnant women and mothers should not be diminished, and more focus on maternal mental health during the epidemic is needed.

## Background

COVID-19, first documented in Wuhan, China at the end of 2019 [1], has rapidly spread across the globe, infecting tens of millions of individuals [2]. While sex-disaggregated data on severe acute respiratory syndrome coronavirus 2 (SARS-CoV-2) mortalities suggest it poses more severe health outcomes for men than women [3], there are concerns that the disease could disproportionately burden women in a social and economic sense. Furthermore, it is a particularly salient question whether pregnant women are more susceptible to infection with SARS-CoV-2 or have more severe disease outcomes. Outside of direct infection, the impact of the pandemic and pandemic-control policies on healthcare infrastructure, societies, and the global economy may also affect maternal health. Pregnant women and new mothers are a unique population, with particular mental and physical healthcare needs who are also particularly vulnerable to issues such as domestic violence. Finally, the impact of the COVID-19 pandemic is likely to be context-specific, and differ depending on a variety of country-specific factors. A global pandemic is likely to only reveal its consequences after significant time passes, and literature published before or immediately after policies are implemented may not capture all relevant outcomes. The goal of this scoping review is to synthesize the current literature on both the direct consequences of contracting COVID-19 during pregnancy and the indirect consequences of the pandemic for pregnant individuals and mothers, taking into account the myriad ways in which containment and prevention measures have disrupted daily life.

## Methods

This scoping review followed the framework outlined by Arksey and O’Malley [4], in order to map the existing literature on the direct and indirect impacts of COVID-19 on maternal health, incorporating the following 5 stages:

### Identify research question

How has the COVID-19 pandemic directly and indirectly impacted maternal health globally?

### Identify relevant types of evidence

Literature published in English from January 1st, 2020 to September 11, 2020 was included in the search. The search strategy involved the algorithm used by the Maternal Health Task Force’s Buzz, a biweekly e-newsletter presenting current research relevant to maternal health. Hand searches were conducted in PubMed using MeSH terms (see Additional file 1), along with broader searches of “COVID” and “corona” followed by the terms: “pregnant”, “maternal”, “women”, “reproductive”, “economic”, “social”, “indirect”, “direct.” Google Scholar was also searched using these terms to capture grey literature, such as news articles and working papers that have not yet completed the peer review process. This scoping review aimed to capture rapidly evolving evidence in a timely manner, including issues not yet addressed in well-funded, epidemiological studies. The snowball method of consulting sources’ bibliographies was used for certain articles to supplement referenced evidence. The search strategy as outlined above was not registered with PROSPERO.

### Study selection

**Fig. 1:**
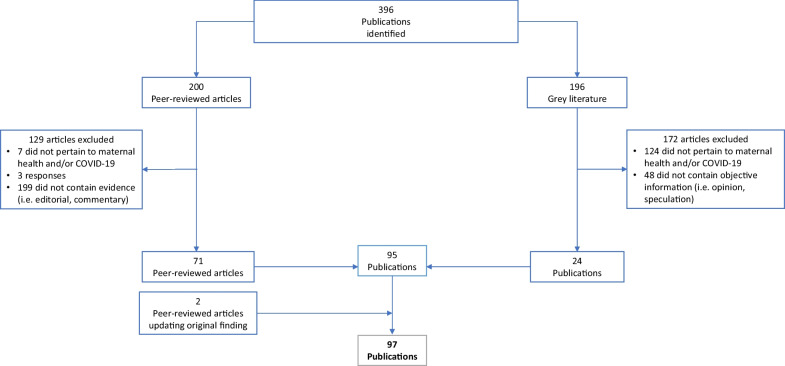
Flowchart of literature selection

Literature was included if published during the time frame outlined above and primarily assessed the direct or indirect effects of the COVID-19 pandemic on maternal health. Search terms utilized did not directly address neonatal health, but publications on topics relevant to both populations (transmission, breastfeeding, maternity care practices) were also included if returned by the search terms. Case reports, case series, qualitative studies, systematic and scoping reviews, and meta-analyses were included. As some publications included were systematic or scoping reviews or meta-analyses, there was some duplication in data on which publications were based. The article containing the more complete description of the data was used for data charting. Sources were excluded if they consisted only of recommendations for future research. Predictive research was excluded if it consisted only of speculation referencing past epidemics but included if based on quantitative methods. News articles, reports, and other grey literature were included if they contained quantifiable evidence (case reports, survey results, qualitative analyses).After reading full texts and synthesizing relevant evidence, literature was organized thematically. Themes were discussed and decided upon by all four authors. Themes that reflected potential impacts of COVID-19, but for which no quantitative evidence existed were excluded from the review. Of 200 peer-reviewed articles, 129 were excluded; 7 did not pertain to maternal health or COVID-19, 3 were responses to articles, and 199 were commentaries, editorials, or practice guidelines which did not contain relevant evidence. Of 196 articles from the grey literature, 172 articles were excluded; 124 did not pertain to maternal health or COVID-19, and 48 did not contain objective information. See Fig. 1 for a visual representation of inclusion and exclusion.

### Chart the data

71 peer-reviewed articles and 24 publications from the grey literature were included from the original search. Two peer-reviewed articles that contradicted earlier findings that were published after September 11, 2020 were added. Publications included represented a wide range of methodologies including case reports, case series, observational studies, letters to the editor, and news articles. The authors developed a rubric of major themes that arose in the literature and recorded standard information including location, sampling method, and size of sample, and key findings of each study (see Table 1). An adaptive thematic analysis [5] was applied using the following steps. The authors identified themes in the literature by a reading and discussing each article included. Articles were then coded independently by two authors. All four authors discussed each code and grouped codes into final themes.

**Table 1:** Studies included in the scoping review

Author, Year	Title	Type of article	Topic	Geographic area of focus	Sample size	Conclusions
Delahoy, September 2020	“Characteristics and Maternal and Birth Outcomes of Hospitalized Pregnant Women with Laboratory-Confirmed COVID-19”	MMWR, population surveillance of 13 states	Direct effects on pregnancy	United States	598	Pregnant women might be at increased risk for severe coronavirus disease
Wu et al. June 2020	“Clinical Manifestation and Laboratory Characteristics of SARS-CoV-2 Infection in Pregnant Women.”	Peer-reviewed, retrospective study	Direct effects on pregnancy	China	8	Close monitoring of laboratory parameters including the WBC count, LYMPH count, and CRP, along with other imaging features in chest CT scans, is warranted to promptly prevent, diagnose, and treat a SARS-CoV-2 infection during pregnancy
Xu et al. April 2020	“Clinical Presentations and Outcomes of SARS-CoV-2 Infected Pneumonia in Pregnant Women and Health Status of Their Neonates.”	Peer reviewed, retrospective study	Direct effects on pregnancy, intrauterine transmission	China	5	No obvious vertical transmission was observed, lymphopenia and eosinopenia were observed more frequently in pregnant COVID-19 patients as compared to pregnant women without COVID-19
Smith et al. June 2020	“Maternal and Neonatal Outcomes Associated with COVID-19 Infection: A Systematic Review.”	Peer reviewed, systematic review	Direct effects on pregnancy, intrauterine transmission, labor and delivery	China	N/A	COVID-19-positive pregnant women present with fewer symptoms than the general population and may be RT-PCR negative despite having signs of viral pneumonia. The incidence of preterm births, low birth weight, C-section, NICU admission appear higher than the general population
Knight et al. June 2020	“Characteristics and Outcomes of Pregnant Women Admitted to Hospital with Confirmed SARS-CoV-2 Infection in UK: National Population Based Cohort Study.”	Peer reviewed, population cohort study	Direct effects on pregnancy, intrauterine transmission	United Kingdom	427	Most pregnant women admitted to hospital with SARS-CoV-2 infection were in the late second or third trimester. Most had good outcomes, and transmission of SARS-CoV-2 to infants was uncommon. The high proportion of women from black or minority ethnic groups admitted with infection needs urgent investigation and explanation
Allotey et al. September 2020	“Clinical Manifestations, Risk factors, and Maternal and Perinatal Outcomes of Coronavirus Disease 2019 in Pregnancy: Living Systematic Review and Meta-analysis”	Peer reviewed, systematic review and meta-analysis	Direct effects on pregnancy	Global	11,432	Pregnant and recently pregnant women are less likely to manifest COVID-19 related symptoms of fever and myalgia than non-pregnant women of reproductive age and are potentially more likely to need intensive care treatment for COVID-19. Pre-existing comorbidities, high maternal age, and high body mass index seem to be risk factors for sever covid-19. Preterm birth rates are high in pregnant women with covid-19 than in pregnant women without the disease
Pereira et al. July 2020	“Clinical Course of Coronavirus Disease-2019 in Pregnancy.”	Peer reviewed, retrospective study	Direct effects on pregnancy, intrauterine transmission, labor and delivery, breastfeeding and infant contact	Spain	60	Most of the pregnant women with COVID‐19 had a favorable clinical course. However, one‐third of them developed pneumonia, of whom 5% presented a critical clinical status. Seventy‐eight percent of the women had a vaginal delivery. No vertical or horizontal transmissions were diagnosed in the neonates during labor or breastfeeding
Blitz et al. June 2020	“Maternal Mortality among Women with Coronavirus Disease 2019 Admitted to the Intensive Care Unit.”	Peer reviewed, case series	Direct effects on pregnancy	New York, United States	462	Maternal death occurred in 15% of patients admitted to the ICUs for COVID-19 and in 25% of those who required invasive mechanical ventilation. Delivery occurred in half of the patients with COVID-19 who were admitted to the ICUs and all patients who required invasive mechanical ventilation. Hispanic women constituted the largest racial or ethnic group in the study, which may reflect a disproportionate burden of disease among minority groups
Chen et al. March 2020	“Clinical Characteristics and Intrauterine Vertical Transmission Potential of COVID-19 Infection in Nine Pregnant Women: A Retrospective Review of Medical Records.”	Peer reviewed, retrospective study	Direct effects on pregnancy, intrauterine transmission, labor and delivery, breastfeeding and infant contact	China	9	The clinical characteristics of COVID-19 pneumonia in pregnant women were similar to those reported for non-pregnant adult patients who developed COVID-19 pneumonia. Findings from this small group of cases suggest that there is currently no evidence for intrauterine infection caused by vertical transmission in women who develop COVID-19 pneumonia in late pregnancy
Yang et al. April 2020	“Clinical Features and Outcomes of Pregnant Women Suspected of Coronavirus Disease 2019.”	Peer reviewed, prospective case control study	Direct effects on pregnancy	China	55	The clinical symptoms and laboratory indicators are not obvious for asymptomatic and mild COVID-19 pregnant women. Pulmonary CT scan plus blood routine examination are more suitable for finding pregnancy women with asymptomatic or mild COVID-19 infection, and can be used screening COVID-19 pregnant women in the outbreak area of COVID-19 infection
Khan et al. March 2020	“Impact of COVID-19 Infection on Pregnancy Outcomes and the Risk of Maternal-to-Neonatal Intrapartum Transmission of COVID-19 during Natural Birth.”	Letter to the editor, case series	Direct effects on pregnancy, labor and delivery	China	3	None of the 3 women in this study had died of COVID-19 infection as of March 1, 2020. No vertical transmission of COVID-19 was found in the third trimester of pregnancy among infants delivered via the vaginal route. Moreover, we did not find evidence of maternal-to-neonatal intrapartum transmission of COVID-19 via vaginal delivery
Lokken et al. May 2020	“Clinical Characteristics of 46 Pregnant Women with a Severe Acute Respiratory Syndrome Coronavirus 2 Infection in Washington State.”	Peer reviewed, retrospective study	Direct effects on pregnancy	Washington, United States	46	Severe coronavirus disease 2019 developed in approximately 15% of pregnant patients and occurred primarily in overweight or obese women with underlying conditions. Obesity and coronavirus disease 2019 may synergistically increase risk for a medically indicated preterm birth to improve maternal pulmonary status in late pregnancy. These findings support categorizing pregnant patients as a higher-risk group, particularly those with chronic comorbidities
Savasi et al. August 2020	“Clinical Findings and Disease Severity in Hospitalized Pregnant Women With Coronavirus Disease 2019 (COVID-19).”	Peer reviewed, prospective cohort study	Direct effects on pregnancy	Italy	77	One in five women hospitalized with COVID-19 infection delivered urgently for respiratory compromise or were admitted to the ICU. None, however, died. Increased pregestational BMI and abnormal heart and respiratory rates on admission were associated with severe disease
Mendoza et al. June 2020	“Pre-Eclampsia-like Syndrome Induced by Severe COVID-19: A Prospective Observational Study.”	Peer reviewed, prospective cohort study	Direct effects on pregnancy	Spain	42	Pregnant women with severe COVID‐19 can develop a PE‐like syndrome that might be distinguished from actual PE by sFlt‐1/PlGF, LDH and UtAPI assessment. Healthcare providers should be aware of its existence and monitor pregnancies with suspected pre‐eclampsia with caution
Ferraiolo et al. June 2020	“Report of Positive Placental Swabs for SARS-CoV-2 in an Asymptomatic Pregnant Woman with COVID-19.”	Peer reviewed, case report	Intrauterine transmission	Italy	1	This was a report of a positive placental swab for SARS-CoV-2 in an asymptomatic woman in the third trimester of pregnancy with a positive rhino-pharyngeal swab for COVID-19, who underwent an urgent caesarian section for obstetric indications
Hosier et al. 2020	“SARS-CoV-2 Infection of the Placenta.”	Peer reviewed, case report	Direct effects on pregnancy, intrauterine transmission	United States	1	This report highlights a case of acute placental infection with SARS–CoV-2 that may have potentiated severe, early-onset preeclampsia
Golden, Thea, & Simmons, July 2020	“Maternal and Neonatal Response to COVID-19.”	Peer reviewed, scoping review	Intrauterine transmission	Global	N/A	At this time, vertical transmission of SARS-CoV-2 is considered unlikely
Huntley et al. August 2020	“Rates of Maternal and Perinatal Mortality and Vertical Transmission in Pregnancies Complicated by Severe Acute Respiratory Syndrome Coronavirus 2 (SARS-Co-V-2) Infection: A Systematic Review.”	Peer reviewed, systematic review	Direct effects on pregnancy, intrauterine transmission, labor and delivery	Global	N/A	There are low rates of maternal and neonatal mortality and vertical transmission with SARS-CoV-2. The preterm birth rate of 20% and the cesarean delivery rate exceeding 80% seems related to geographic practice patterns
Walker et al. June 2020	“Maternal Transmission of SARS-COV-2 to the Neonate, and Possible Routes for Such Transmission: A Systematic Review and Critical Analysis.”	Peer reviewed, systematic review	Intrauterine transmission, labor and delivery, breastfeeding and infant contact	Global	655	Neonatal COVID‐19 infection is uncommon, rarely symptomatic, and the rate of infection is no greater when the baby is born vaginally, breastfed or remains with the mother
Zhang et al. July 2020	“Severe Acute Respiratory Syndrome Coronavirus 2 (SARS-CoV-2) Infection During Late Pregnancy: A Report of 18 Patients from Wuhan, China	Peer reviewed, case series	Direct effects on pregnancy, intrauterine transmission, labor and delivery	China	18	The majority of patients in late term pregnancy with COVID-19 were of ordinary type, and they were less likely to develop into critical pneumonia after early isolation and antiviral treatment. Vertical transmission of SARS-CoV-2 was not detected, but the proportion of neonatal bacterial pneumonia was higher than other neonatal diseases in newborns
Martins-Filho et al. April 2020	“To Breastfeed or Not to Breastfeed? Lack of Evidence on the Presence of SARS-CoV-2 in Breastmilk of Pregnant Women with COVID-19.”	Peer reviewed, rapid systematic review	Breastfeeding and infant contact	Global	N/A	No breast milk samples were positive for SARS-CoV-2 and, to date, there is no evidence on the presence of SARS-CoV-2 in breast milk of pregnant women with COVID-19
Perrone et al. May 2020	“Lack of Viral Transmission to Preterm Newborn from a COVID-19 Positive Breastfeeding Mother at 11 Days Postpartum.”	Peer reviewed, case report	Breastfeeding and infant contact	Italy	1	During a stay in the hospital, mother and healthcare caregivers applied recommended hygiene measures, consisting of wearing surgical‐mask, hand washing, and using alcohol‐based solutions to clean the surfaces. In this setting, no horizontal transmission occurred. Moreover, RT‐PCR assay for SARS‐CoV‐2 performed on breast milk during mother febrile peak was negative. This is in line with recent studies, RT‐PCR assays on breast milk samples collected from affected women result negative for SARS‐CoV‐2
Zeng et al. March 2020	“Antibodies in Infants Born to Mothers With COVID-19 Pneumonia.”	Peer reviewed, research letter	Breastfeeding and infant contact	China	6	Among 6 mothers with confirmed COVID-19, SARS-CoV-19 was not detected in the serum or throat swab by RT-PCR in any of their newborns. However, virus-specific antibodies were detected in neonatal blood sera samples
Dong et al. May 2020	“Possible Vertical Transmission of SARS-CoV-2 From an Infected Mother to Her Newborn.”	Peer reviewed, research letter	Intrauterine transmission, breastfeeding and infant contact	China	1	A neonate born to a mother with COVID-19 had elevated antibody levels and abnormal cytokine test results 2 h after birth. The elevated IgM antibody level suggests that the neonate was infected in utero
Kimberlin, David & Stagno, March 2020	“Can SARS-CoV-2 Infection Be Acquired In Utero?: More Definitive Evidence Is Needed.”	Peer reviewed, editorial	Intrauterine transmission, breastfeeding and infant contact	Global	N/A	More definitive evidence is needed before concluding that fetuses are at risk from congenital infection with SARS-CoV-2
Egloff et al. July 2020	“Evidence and Possible Mechanisms of Rare Maternal–Fetal Transmission of SARS-CoV-2.”	Peer reviewed, review	Intrauterine transmission	France	179	Among 179 newborns tested for SARS-CoV2 at birth from mothers with COVID-19, transmission was suspected in 8 cases, 5 with positive nasopharyngeal SARS-CoV-2 RT-PCR and 3 with SARS-CoV-2 IgM
Wu et al. May 2020	“Coronavirus Disease 2019 among Pregnant Chinese Women: Case Series Data on the Safety of Vaginal Birth and Breastfeeding.”	Peer reviewed, single center cohort study	Intrauterine transmission, breastfeeding	China	13	Vaginal delivery may be a safe delivery option. However, additional research is urgently needed to examine breast milk and the potential risk for viral contamination
Ashokka et al. July 2020	“Care of the Pregnant Woman with Coronavirus Disease 2019 in Labor and Delivery: Anesthesia, Emergency Cesarean Delivery, Differential Diagnosis in the Acutely Ill Parturient, Care of the Newborn, and Protection of the Healthcare Personnel.”	Peer reviewed, clinical opinion	Labor and delivery	Global	N/A	We present management strategies derived from best available evidence to provide guidance in caring for the high-risk and acutely ill parturient
Oxford-Horrey et al. August 2020	“Putting It All Together: Clinical Considerations in the Care of Critically Ill Obstetric Patients with COVID-19.”	Peer reviewed, review	Labor and delivery	U.S	N/A	Guidelines from various clinical societies, along with direction from local health authorities, must be considered when approaching the care of an obstetric patient with known or suspected COVID-19. With a rapidly changing landscape, a simplified and cohesive perspective using guidance from different clinical society recommendations regarding the critically-ill obstetric patient with COVID-19 is needed
Gatta, Nunzia, Rizzo, Pilu & Simonazzi, April 2020	“COVID19 during Pregnancy: A Systematic Review of Reported Cases.”	Peer reviewed, systematic review	Labor and delivery	Global	51	At the time of the report, 3 pregnancies were ongoing; of the remaining 48 pregnant women, 46 gave birth by cesarean delivery, and 2 gave birth vaginally
Zaigham & Andersson, April 2020	“Maternal and perinatal outcomes with COVID-19: A systematic review of 108 pregnancies.”	Peer reviewed, systematic review	Labor and delivery	Global	108	91% of the women were delivered by cesarean section
Chen et al. April 2020	“Clinical Characteristics of Pregnant Women with Covid-19 in Wuhan, China.”	Letter to the editor, review	Labor and delivery	China	118	Of the 68 patients who delivered during the study period, 63 (93%) underwent a cesarean section
Malhotra et al. June 2020	“No Change in Cesarean Section Rate during COVID-19 Pandemic in New York City.”	Letter to the editor	Labor and delivery	U.S	N/A	We found that there were no changes in Cesarean section rate during the COVID-19 pandemic in New York City
COVIDSurg Collaborative, May 2020	“Elective surgery cancellations due to the COVID-19 pandemic: Global predictive modelling to inform surgical recovery plans”	Peer reviewed, modelling study	Labor and delivery	Global	N/A	Globally, 81.7 per cent of operations for benign conditions,37.7 per cent of cancer operations and 25.4 per cent of elective caesarean sections would be cancelled or postponed
Narang et al. May 2020	“SARS-CoV-2 in Pregnancy: A Comprehensive Summary of Current Guidelines.”	Peer reviewed, summary of guidelines	Labor and delivery	Global	N/A	The summary of guidelines for the management of COVID-19 in pregnancy across different perinatal societies is fairly consistent, with some variation in the strength of recommendations
Lei et al. March 2020	“Clinical Characteristics of COVID-19 in Pregnancy: Analysis of Nine Cases.”	Retrospective case study	Breastfeeding and infant contact	China	9	Serial real-time quantitative reverse transcription-polymerase chain reaction showed negative results in the detection of 2019-novel coronavirus in all samples obtained from amniotic fluid, umbilical cord blood, neonatal nasopharynx, breast milk, and vagina
Wang et al. July 2020	“A Case Report of Neonatal 2019 Coronavirus Disease in China.”	Peer reviewed, case study	Breastfeeding and infant contact	China	1	The mother’s breast milk sample was negative for SARS-CoV-2 as well
Zhu et al. June 2020	“Breastfeeding Risk from Detectable Severe Acute Respiratory Syndrome Coronavirus 2 in Breastmilk.”	Peer reviewed, retrospective case study	Breastfeeding and infant contact	China	5	Four out of five (80%) patient`s breastmilk samples were negative for SARS-CoV-2 RT-PCR, which is similar to previous observations,2,8 while one (20%) patient`s (Patient 3) breastmilk showed SARS-CoV-2 RNA test positive
Dong et al. January 2020	“Antibodies in the Breast Milk of a Maternal Woman with COVID-19.”	Peer reviewed, case study	Breastfeeding and infant contact	China	1	A maternal woman was positive for SARS-CoV-2 tested in throat swabs but negative tested in other body fluids, and she had IgG and IgA detected in breast milk
Bastug et al. July 2020	“Virolactia in an Asymptomatic Mother with COVID-19.”	Peer reviewed, case study	Breastfeeding and infant contact	Turkey	1	Temporary separation of the newborn from a mother with confirmed or suspected COVID-19 should be strongly considered to reduce the risk of transmission to the neonate
Wu et al. August 2020	“Perinatal Depressive and Anxiety Symptoms of Pregnant Women during the Coronavirus Disease 2019 Outbreak in China.”	Peer reviewed, multicenter, cross-sectional study	Mental health	China	4124	Pregnant women assessed after the declaration of coronavirus disease 2019 epidemic had significantly higher rates of depressive symptoms than women assessed before the epidemic declaration. These women were also more likely to have thoughts of self-harm. The depressive rates were positively associated with the number of newly confirmed cases of coronavirus disease 2019, suspected infections, and deaths per day
Saccone et al. May 2020	“Psychological Impact of Coronavirus Disease 2019 in Pregnant Women.”	Peer reviewed, cross-sectional survey study	Mental health	Italy	100	The COVID-19 outbreak had a moderate to severe psychological impact on pregnant women
Jungari, June 2020	“Maternal Mental Health in India during COVID-19.”	Peer reviewed, letter to the editor	Mental health	India	N/A	Pregnant women and new mothers are at an elevated risk of suffering from mental health problems. It has been observed that the uncertainty surrounding COVID-19 has led to higher levels of depression among women during and after pregnancy
Gausman & Langer, April 2020	“Sex and Gender Disparities in the COVID-19 Pandemic.”	Peer reviewed, commentary	Mental health	Global	N/A	Although containment strategies … may be clinically important to reduce transmission, they may also have profound short- and long-term mental health implications for women
Aryal & Pant, December 2020	“Maternal Mental Health in Nepal and Its Prioritization during COVID-19 Pandemic: Missing the Obvious.”	Peer reviewed, letter to the editor	Mental health	Nepal	N/A	Lack of counseling, uncertainty and indecisiveness increases stress during pregnancy. In addition to this, pregnant women are worrying about COVID19 effects on their health and their newborns
Kotabagi, Fortune, Essien, Nauta, & Yoong, July 2020	“Anxiety and Depression Levels among Pregnant Women with COVID-19.”	Peer reviewed, letter to the editor	Mental health	UK	N/A	Median score rose to a maximum at the height of the pandemic deaths in the UK when “lockdown” rules were instituted amid great uncertainty about National Health Service capacity and COVID outcomes
Chivers et al. Septermber 2020	“Perinatal Distress During COVID-19: Thematic Analysis of an Online Parenting Forum”	Peer reviewed, qualitative thematic analysis	Mental health	Australia	N/A	Themes were (1) heightened distress related to a high-risk external environment; (2) despair and anticipatory grief due to deprivation of social and family support, and bonding rituals; (3) altered family and support relationships; (4) guilt-tampered happiness; and (5) family future postponed
Koenen, July 2020	“Pregnant During a Pandemic?”	Blog article	Mental health	U.S	N/A	Over 70% of women report clinically significant depression or anxiety, and over 40% screen positive for post-traumatic stress disorder (PTSD)
Thapa, Mainali, Schwank, & Acharya, July 2020	“Maternal Mental Health in the Time of the COVID-19 Pandemic.”	Peer reviewed, editorial	Mental health	Global	N/A	Strict public health measures directed towards mitigating the spread of disease are necessary but known to have negative psychological effects leading to stress, anger and confusion
Preis et al. December 2020	“Vulnerability and resilience to pandemic-related stress among U.S. women pregnant at the start of the COVID-19 pandemic”	Peer reviewed, survey	Mental health, Prenatal care	U.S	4451	Nearly one-third of pregnant women highly stressed by the COVID-19 pandemic
Roberton et al. May 2020	“Early estimates of the indirect effects of the COVID-19 pandemic on maternal and child mortality in low-income and middle-income countries: a modelling study”	Peer reviewed, modelling study	Prenatal care	Global	N/A	Model estimates a reduction in antenatal care by at least 18%, and possibly up to 51.9%, and a similar reduction in postnatal care
Menendez et al. May 2020	“Avoiding Indirect Effects of COVID-19 on Maternal and Child Health.”	Peer reviewed, comment	Prenatal care	Global	N/A	In the context of the COVID-19 pandemic, some African countries are changing routine ANC guidelines to space (and de facto reduce) the number of contacts to one every 3 months instead of monthly visits, or delaying the postpartum visit to 3 months after delivery (therefore no longer constituting a postpartum visit)
Ramoni, June 2020	“How COVID-19 Is Affecting Antenatal Care.”	News article	Prenatal care	Nigeria	N/A	Dr. Adeyemi Okunowo, admitted that with COVID-19, the normal way of caring for pregnant women had been modified to reduce the spread of the disease
Aryal, Shrestha, May 2020	“Motherhood in Nepal during COVID-19 Pandemic: Are We Heading from Safe to Unsafe?”	Comment	Prenatal care	Nepal	N/A	There is a possibility of delay in seeking care when pregnant mothers are unsure when to visit the hospitals because of the uncertainty of availability of their services during the pandemic. For women hailing from remote areas, the travel ban during the lockdown causes a delay in reaching care
Pallangyo et al. June 2020	“The Impact of Covid-19 on Midwives’ Practice in Kenya, Uganda and Tanzania: A Reflective Account.”	Peer reviewed, reflective account	Prenatal care	Kenya, Uganda, and Tanzania	N/A	Midwives have reported low numbers attending maternal health clinics and women are afraid to visit the hospitals for fear of contracting coronavirus… This has led to women coming into hospitals too late, sometimes ending with undesirable outcomes e.g. stillbirths, neonatal and maternal death
Population Council, April 2020	“Kenya: COVID-19 Knowledge, Attitudes, Practices and Needs—Responses from Second Round of Data Collection in Five Nairobi Informal Settlements (Kibera, Huruma, Kariobangi, Dandora, Mathare).”	Survey	Prenatal care, gender equity in the workforce	Kenya	1,769	More women reported complete loss of income/employment compared to men. Women were twice as likely to forgo essential health services, including family planning services
ReliefWeb, April 2020	“Rapid Gender Analysis—COVID-19: West Africa—April 2020—Benin.”	Analysis	Prenatal care	West Africa	266	Between social distancing slowing down all service provisions and the fear of attending clinics, it is very hard for women to access SRHR services. Women and youth have little access to traditional information channels like TV and radio because men control these outlets in the household
Semaan et al. June 2020	“Voices from the Frontline: Findings from a Thematic Analysis of a Rapid Online Global Survey of Maternal and Newborn Health Professionals Facing the COVID-19 Pandemic.”	Peer reviewed, global, cross-sectional study	Prenatal care	Global	714	Healthcare providers are worried about the impact of rapidly changing care practices on health outcomes: reduced access to antenatal care, fewer outpatient visits, shorter length of stay in facilities after birth, banning birth companions, separating newborns from COVID-19 positive mothers and postponing routine immunizations
Coxon et al. September 2020	“The Impact of the Coronavirus (COVID-19) Pandemic on Maternity Care in Europe.”	Peer reviewed, editorial	Prenatal care	U.K	N/A	Concern about what constitutes safe care of pregnant women and newborns has increased, and in many settings, risk averse decisions have been taken in maternity care provision which, it is argued, may increase unnecessary medical interventions, put women at risk of being infected with COVID-19 by reducing provision of community or home-based care, and reduce or reverse progression towards high quality maternity care
Ayenew et al. June 2020	“Risk for Surge Maternal Mortality and Morbidity during the Ongoing Corona Virus Pandemic.”	Preprint	Prenatal care	India and Ethiopia	N/A	Lockdown and quarantine secondary to COVID-19 pandemic could have serious consequences for women's health more than ever. Poor accessibility and low literacy rate to use it is another challenge to implementation telemedicine in developing countries
Stevis-Gridneff, Haridasani Gupta, Monica Pronczuk	“Coronavirus Created an Obstacle Course for Safe Abortions.”	News article	Healthcare Infrastructure	Europe	N/A	For many women in Europe seeking abortions, the COVID-19 pandemic added another obstacle in an already complicated and time-sensitive course
Frederisken, June 2020	“State Action to Limit Abortion Access During the COVID-19 Pandemic.”	News article	Healthcare Infrastructure	U.S	N/A	The response to the COVID-19 pandemic has prompted several states to place restrictions that have effectively banned or blocked the availability of abortion services
UNFPA, April 2020	“Impact of the COVID-19 Pandemic on Family Planning and Ending Gender-Based Violence, Female Genital Mutilation and Child Marriage.”	Report	Healthcare Infrastructure	Global	N/A	If the lockdown continues for 6 months and there are major service disruptions due to COVID-19, an additional 7 million unintended pregnancies are expected to occur
International Planned Parenthood Federation, April 2020	“COVID-19 Pandemic Cuts Access to Sexual and Reproductive Healthcare for Women around the World”	News article	Healthcare Infrastructure	Global	N/A	5,633 static and mobile clinics and community-based care outlets have already closed because of the outbreak, across 64 countries. They make up 14% of the total service delivery points IPPF members ran in 2018
Gettleman & Raj, July 2020	“8 Hospitals in 15 h: A Pregnant Woman’s Crisis in the Pandemic.”	News article	Healthcare Infrastructure	India	N/A	Her baby was coming, and her complications were growing more dangerous. But nowhere would take her — an increasingly common story as India’s health care system buckles under pressure
Kumari, Mehta, & Choudhary, July 2020	“COVID-19 outbreak and decreased hospitalisation of pregnant women in labour.”	Peer reviewed, retrospective analysis	Healthcare Infrastructure	India	N/A	Our initial analysis of women admitted during the lockdown period revealed a 43·2% reduction in hospitalisation compared with the control period and a 49.8% reduction compared with the same calendar period from the previous year. Referred obstetric emergencies also decreased by 66.4%
Takemoto et al. July 2020	“Maternal Mortality and COVID-19.”	Peer reviewed, systematic review	Healthcare Infrastructure	Brazil	20	Barriers to access healthcare, differences in pandemic containment measures in the country and high prevalence of concomitant risk factors for COVID-19 severe disease may play a role in the observed disparity compared to worldwide reports on maternal outcomes
Rafaeli & Hutchinson, June 2020	“The Secondary Impacts of COVID-19 on Women and Girls in Sub-Saharan Africa”	Preprint, rapid review	Healthcare Infrastructure	Sub-Saharan Africa	N/A	There is strong evidence to suggest that women and girls in SSA will suffer from extreme and multifaceted negative secondary impact as a result of the COVID-19 crisis
Khalil et al. July 2020	“Change in the Incidence of Stillbirth and Preterm Delivery During the COVID-19 Pandemic”	Peer reviewed, retrospective study	Healthcare Infrastructure	U.K	3399	The incidence of stillbirth was significantly higher during the pandemic period (none associated with COVID-19) than during the prepandemic period
KC et al. August 2020	“Effect of the COVID-19 pandemic response on intrapartum care, stillbirth, and neonatal mortality outcomes in Nepal: A prospective observational study”	Peer reviewed, prospective observational study	Healthcare Infrastructure	Nepal	21,763	The institutional stillbirth rate increased from 14 per 1000 total births before lockdown to 21 per 1000 total births during lockdown, and institutional neonatal mortality increased from 13 per 1000 livebirths to 40 per 1000 livebirths
Shuchman, May 2020	“Low- and Middle-Income Countries Face up to COVID-19.”	News article	Healthcare Infrastructure	Global	N/A	On and off the front lines, doctors are watching in alarm as COVID-19 hits areas with vulnerable populations and fragile healthcare systems. Peter Hotez, Dean of the National School of Tropical Medicine at Baylor College of Medicine in Houston, Texas, expects the pandemic to exact a bigger toll in the Global South than in North America or Europe, for several reasons
Bong et al. April, 2020	“The COVID-19 Pandemic: Effects on Low- and Middle-Income Countries.”	Peer reviewed, research summary	Healthcare infrastructure	Global	N/A	In the absence of specific, effective treatment and given a lack of resources in managing active COVID-19 patients, prevention and early containment of the disease appear to be the most feasible option for LMICs
Hupaku & Petrongolo, 2020	“COVID-19 and Gender Gaps: Latest Evidence and Lessons from the UK.”	Peer reviewed, research-based policy analysis and commentary	Gender equity in the workforce	United Kingdom	N/A	The social distancing and lockdowns associated with the COVID-19 crisis has hit service sectors with frequent interactions between consumers and providers which cannot be done from home. At the same time, it has added education and childcare services to pre-existing home production needs
Robertson & Gebeloff, April, 2020	“How Millions of Women Became the Most Essential Workers in America.”	Newspaper article	Gender equity in the workforce	United States	N/A	Though deemed “essential workers” during the COVID-19 pandemic, female healthcare workers face inequality with regards to wages, resource allocation, and general respect and prestige compared to their male counterparts
Jankowski et al. May, 2020	“Risk Stratification for Healthcare Workers during CoVID-19 Pandemic: Using Demographics, Co-Morbid Disease and Clinical Domain in Order to Assign Clinical Duties.”	Preprint literature review	Gender equity in the workforce	Global	N/A	We have generated a tool which can provide a framework for objective risk stratification of doctors and health care professionals during the COVID-19 pandemic
World Health Organization, March, 2020	“Shortage of Personal Protective Equipment Endangering Health Workers Worldwide.”	News release	Gender equity in the workforce	Global	N/A	To meet rising global demand for personal protective equipment (PPE), WHO estimates that industry must increase manufacturing by 40 per cent
Alon et al. 2020	“The Impact of COVID-19 on Gender Equality.”	Working paper	Gender equity in the workforce	United States	N/A	The economic downturn caused by the current COVID-19 outbreak has substantial implications for gender equality, both during the downturn and the subsequent recovery
Johnston, Mohammed, & van der Linden, 2020	“Evidence of Exacerbated Gender Inequality in Child Care Obligations in Canada and Australia During the COVID-19 Pandemic.”	Peer reviewed, research article	Gender equity in the workforce	Canada & Australia	7746	Households in Canada and Australia have exhibited similar trends in the gendered allocation of additional child care responsibilities resulting from policy responses to the COVID-19 pandemic
Malik & Naeem, 2020	“Impact of COVID-19 Pandemic on Women: Health, livelihoods & domestic violence.”	Peer reviewed, policy review	Gender equity in the workforce	Pakistan	N/A	The government should map out a plan of action to counter the short and long-term effects of the coronavirus on women keeping in view their health, livelihoods, and domestic violence
UN Women, May, 2020	The Private Sector's Role in Mitigating the Impact of COVID-19 on Vulnerable Women and Girls in Nigeria	Peer reviewed, research brief	Gender equity in the workforce	Eastern and Southern Africa	N/A	The Nigerian private sector has assumed a leading role in the fight against the COVID-19 pandemic in the country. CACOVID aimed to also help mobilize private sector thought leadership, raise public awareness and buy-in for COVID-19 prevention, and provide direct support to strengthen the healthcare sector’s capacity to respond to the crisis
Wahome, April, 2020	“Impact of Covid-19 on Women Workers in the Horticulture Sector in Kenya. *Hivos.”*	Peer reviewed, action-oriented rapid assessment summary report	Gender equity in the workforce	Nairobi	71	The impact of Covid-19 pandemic has impacted women workers in the horticulture sector socially, economically and psychologically, with the effect spiraling to their homes
World Vision International Cambodia, June, 2020	“Rapid Assessment of the impact of COVID-19 on child wellbeing in Cambodia Summary Report.”	Peer reviewed, rapid assessment summary report	Gender equity in the workforce	Cambodia	222 households, 42 key informant interviews, 65 agricultural cooperative leaders	The COVID-19 outbreak is already having a severe impact on livelihood, food security, and education, especially among the most vulnerable families in Cambodia
Staff of the National Estimates Branch, July, 2020	“Current Employment Statistics Highlights.”	Peer reviewed, detailed industry employment analysis	Gender equity in the workforce	United States	N/A	During the time period of the COVID-19 pandemic, different industries saw both increases and decreases in employment
Adams-Prassl, et al. April, 2020	“Inequality in the Impact of the Coronavirus Shock: Evidence from Real Time Surveys. IZA Discussion Papers.”	Discussion paper	Gender equity in the workforce	United Kingdom, United States, and Germany	20,910	The labor market impacts of COVID-19 differ considerably across countries. Within countries, the impacts are highly unequal and exacerbate existing inequalities. Workers in alternative work arrangements and in occupations in which only a small share of tasks can be done from home are more likely to have reduced their hours, lost their jobs and suffered falls in earnings. Less educated workers and women are more affected by the crisis
Viveiros & Bonomi, June, 2020	“Novel Coronavirus (COVID-19): Violence, Reproductive Rights and Related Health Risks for Women, Opportunities for Practice Innovation”	Peer reviewed, commentary	Domestic violence	Global		Now is the time for violence prevention leaders to advocate for bold action. This includes prioritizing the needs of women (especially minoritized women) in medical, social and legal settings using innovative intervention and service engagement (e.g., e-filing for protection orders, virtual advocacy services), urging policy makers to pass legislation to support women, and shining an accountability spotlight on leadership
Wanqing, March, 2020	“Domestic Violence Cases Surge During COVID-19 Epidemic.”	Magazine article	Domestic violence	China	N/A	Rates of domestic violence in China increased during the COVID-19 pandemic, many of which were attributable to the epidemic itself, but due to the focus on the pandemic, the needs of victims were neglected
Euronews, March, 2020	“Domestic Violence Cases Jump 30% during Lockdown in France.”	News article	Domestic violence	France	N/A	Domestic violence cases in France increased following the onset of a nationwide lockdown due to COVID-19
UN Women, April, 2020	“COVID-19 and Ending Violence against Women and Girls*.*”	Issue brief	Domestic violence	Global	N/A	It is critical to address the increase of violence against women during COVID-19 through accelerated and concerted e orts of governments, international and national civil society organizations and UN agencies. The needs of women and girls who face multiple forms of discrimination need to be prioritized
Sigal, et al. April, 2020	“‘Another Pandemic’: In Latin America, Domestic Abuse Rises amid Lockdown.”	News article	Domestic violence	Latin America	N/A	Lockdowns around Latin America are helping slow the spread of COVID-19, but are having a darker and less-intended consequence: a spike in calls to helplines suggests a rise in domestic abuse, in a region where almost 20 million women and girls suffer sexual and physical violence each year
Graham-Harrison, et al. March, 2020	“Lockdowns around the World Bring Rise in Domestic Violence.”	News article	Domestic violence	Global	N/A	Domestic violence against women and children has increased as a result of the quarantine during the COVID-19 pandemic
Bosman, August, 2020	“Domestic Violence Calls Mount as Restrictions Linger: ‘No One Can Leave.’”	Newspaper article	Domestic violence	United States	N/A	The coronavirus has created new tensions. Staying at home has worsened abusive situations. Shelters worry about the spread of the virus
Women’s Safety NSW, April, 2020	“New Domestic Violence Survey in NSW Shows Impact of COVID-19 on the Rise.”	Peer reviewed, media release	Domestic violence	Australia	80	Surveys of frontline women’s domestic violence workers during the COVID-19 pandemic reveal increased overall clients utilizing their services, increased complexity of client needs, escalated or worsened violence, increased violence specifically relating to COVID-19, and increased violence reported for the first time
Ndedi, April, 2020	“Framework in Ending Violence Against women and Girls with the Advent of the COVID 19 from an African Perspective.”	Evidence-based framework	Domestic violence	Africa	N/A	There is a need to increase of knowledge and awareness-support for community mobilization. It is also important to engage with telecom mobile companies to deliver messages and provide services like interventions challenging violent masculinities and CSOs advocacy on ending gender-based violence with the COVID-19. Finally, the provision of quality essential services adapted to the current pandemic context is essential
Mukherjee et al. June, 2020	“Vulnerability of Major Indian States Due to COVID-19 Spread and Lockdown.”	Analysis of state- and national- level health data sources	Domestic violence	India	N/A	There are many states in India, including Gujarat, that have a higher percentage of women experiencing domestic violence with drunkard husband as compared to the national average. The lockdown is likely to affect these sections of women much adversely as they are locked at home with their abusers who are likely to get more violent in the absence of liquor. The National Commission for Women (NCW) on 17th April, 2020 said it registered 587 domestic violence complaints between March 23 and April 16—a significant surge from 396 complaints received in the previous 25 days between February 27 and March 22
DeBolt et al. November 2020_a_	“Pregnant women with severe or critical coronavirus disease 2019 have increased composite morbidity compared with nonpregnant matched controls.”	Peer reviewed, multicenter, retrospective, case–control study	Direct effects on pregnancy	38	U.S	Pregnant women with severe and critical coronavirus disease 2019 are at an increased risk for certain morbidities when compared with nonpregnant controls
Zambrano et al. October 2020_a_	“Update: Characteristics of Symptomatic Women of Reproductive Age with Laboratory-Confirmed SARS-CoV-2 Infection by Pregnancy Status—United States, January 22–October 3, 2020”	MMWR population, surveillance	Direct effects on pregnancy	400,000	U.S	In an analysis of approximately 400,000 women aged 15–44 years with symptomatic COVID-19, intensive care unit admission, invasive ventilation, extracorporeal membrane oxygenation, and death were more likely in pregnant women than in nonpregnant women

_a_ Study added in after September 11 to reflect updates in the knowledge.

### Collate, summarize, and report results

Narrative descriptions of the evidence were written for each theme that the authors determined in the above stages. All authors reviewed descriptions for clarity and relevance and some themes were combined post hoc to improve readability and avoid redundancy.

## Main text

### Direct effects on pregnancy

During pregnancy, people undergo significant physiologic and immunologic alterations to support and protect the developing fetus. These changes can increase the risk of infection with respiratory viruses for pregnant individuals and their fetuses. Thus, pregnant individuals and their children may be at heightened risk for infection with SARS-CoV-2 [2].

In general, pregnant individuals with COVID-19 do not seem to display more severe disease symptoms than non-pregnant individuals. Most cases among pregnant people are asymptomatic or mildly symptomatic [6]. For symptomatic cases, the most common clinical presentations included fever, cough, and dyspnea [7–11]. Laboratory findings consistently included lymphopenia, leukopenia, thrombocytopenia, and elevated levels of C-reactive protein and transaminases [7, 12–14]. Others reported an increased D-dimer level and neutrophil/lymphocyte ratio and a decreased white blood cell count [8, 9]. Chest computed tomography (CT) scans revealed abnormal imaging features, namely ground-glass opacities, in the lungs of pregnant individuals with COVID-19 [7, 10, 15], but the clinical significance of these imaging findings and the laboratory parameters is not clear.

Adverse outcomes resulting from maternal infection with SARS-CoV-2 during pregnancy are infrequent. In studies from January to September 2020, most cases of COVID-19 among pregnant individuals documented during surveillance in the United States did not progress to severe disease, and intensive care unit (ICU) admission involving mechanical ventilation was seldom required [6]. Results were similar in two studies of pregnant women admitted hospitals in China [7, 16]. However, recently two studies have contradicted these early results. A multicenter, retrospective case control study published in November 2020 compared pregnant women admitted in Philadelphia for severe or critical coronavirus disease to reproductive-aged nonpregnant women admitted for severe or critical coronavirus disease found that pregnant participants were more likely to be admitted to the ICU, to be intubated, to require mechanical ventilation, and were at increased risk of composite morbidity [17]. Similarly, an analysis of 400,000 women in the United States between 15 and 44 years of age with symptomatic COVID-19 published in October 2020 found that pregnant women were more likely to experience ICU admission, intubation, mechanical ventilation, and death [18].

Publications including data from a variety of contexts and designs found that the most commonly reported adverse outcome was preterm delivery [11–13, 19]; and increased prevalence of low birthweight and Cesarean-section (C-section) delivery were also observed [10, 20]. Other obstetric complications and outcomes including maternal death, stillbirth, miscarriage, preeclampsia, fetal growth restriction, coagulopathy, and premature rupture of membranes were rare, but apparent [8]. Epidemiological studies did not show that COVID-19 directly increased risks for these outcomes, although a study in London [21] suggests that stillbirths may become more common as a direct or indirect consequence of the pandemic. A prospective cohort study by Mendoza et al. found that pregnant individuals with severe COVID-19 may develop a preeclampsia-like syndrome without abnormal ratios of soluble fms-like tyrosine kinase 1 to placental growth factor (sFlt-1/PIGF) and uterine artery pulsatile index (UtAPI) scores typical of normal preeclampsia [22]. Placental infection with the virus was observed; however, these cases were largely asymptomatic or mildly symptomatic [23, 24]. In a review of cases, Golden and Simmons hypothesized that these placental abnormalities were not a direct result of COVID-19 infection [25].

### Intrauterine transmission

The literature on maternal–fetal transmission of SARS-CoV-2 is highly speculative and requires additional evidence to confirm postulated mechanisms of transmission. Thus far, studies do not support intrauterine infection with COVID-19 resulting from vertical transmission in pregnant individuals with clinically or microbiologically diagnosed cases of the virus during the third trimester [14, 26–28]. Few cases of neonatal infection potentially acquired in utero were observed. For example, samples from six pregnant women with COVID-19 and their neonates in Wuhan, China were tested, and SARS-CoV-2 RNA was undetectable in samples of cord blood, throat and nasopharyngeal swabs, urine, feces, amniotic fluid, and placental tissue [14, 29, 30]. Yet, the reliability of these positive neonatal test results was questioned as tests were not performed immediately following delivery. Elevated IgM antibodies in neonates with SARS-CoV-2 infection born to SARS-CoV-2-positive mothers were identified at two hospitals in Wuhan, China [31, 32] However, Kimberlin and Stagno raised doubts about intrauterine transmission as IgM antibodies are too large to cross the placenta. Also, IgM assays used to diagnose congenital infection are often unreliable [33]. Low levels of SARS-CoV-2 RNA were found in blood samples collected in two large cohort studies in Wuhan, China [34, 35]. The occurrence of placental infection with the virus [23, 24] and the presence of COVID-19 antibodies in neonatal blood suggest some mechanism of vertical transmission [25].

Despite this, transmission of the virus through blood is questionable. Egloff et al. hypothesized that transcytosis (transcellular transport) of the virus, infected blood cell transport, and virus or infected cells in the cervicovaginal compartment were unlikely avenues for transmission in most pregnant women with COVID-19. According to these data, the maternal–fetal transmission risk is probably very low, possibly under 1% following maternal SARS-CoV-2 infection during pregnancy [36]. Yet, it is widely recognized that further research involving larger population-based longitudinal studies is needed to determine the plausibility of incidental maternal–fetal transmission.

### Labor and delivery

A few case series including an analysis of 108 births occurring in New York City, suggest no increased risk of infection for the neonate when birth occurs vaginally. Despite early reassuring evidence that there is no increased risk of infection for the neonate when birth occurs vaginally [37], clinical guidelines differ in their recommendations on mode of delivery [38, 39].

Estimates of C-section rates among women infected with SARS-CoV-2 differed but suggest a potentially significant increase in operative delivery. A systematic review conducted by Della Gatta et al. reported that 90.2% of women diagnosed with COVID-19 delivered via C-section [40] A systematic review by Zaigham and Andersson reported 91% of the women delivered via C-section [41] This is similar to early estimates from Wuhan, China; Chen et al. found a C-section occurrence of 93% [42]. The reasons for this practice are unclear, but it may be attributable to more aggressive management of labor and delivery during the onset of the pandemic. However, a recent analysis of women delivering at New York City hospitals between March 8 and April 2, 2020 found C-section rates not higher than average (31.3% for women with confirmed COVID-19, compared to 33.9% of those who tested negative) [43]. Some scientists and healthcare providers speculated that C-section rates are reduced in LMICs due to indirect impacts of the COVID-19 pandemic on the healthcare system [44]. As of the completion of this paper, no evidence to support this exists.

Particularly in the beginning of the pandemic, hospitals implemented policies regarding support persons and postpartum stays that isolated women during labor and delivery. A comprehensive review of care guidelines from international perinatal societies and institutions found that most either recommended no visitors or one asymptomatic support person, and expedited discharge was recommended by the American College of Obstetrics and Gynecology, the Catalan Health Service, and the Society for Maternal and Fetal Medicine [45]. Given the documented benefits of labor support [46], reducing access may increase the incidence of C-section delivery and decrease maternal satisfaction with labor and delivery experiences. Furthermore, expedited discharge may reduce the ability of healthcare providers to identify and treat postpartum complications.

### Breastfeeding and infant contact

The possibility of transmission of novel coronavirus through breast milk is unclear. The published evidence on the presence of SARS-CoV-2 in breastmilk consisted of case reports and case series of postpartum women who tested positive for the coronavirus during pregnancy. Of milk samples collected from 37 women, the majority tested negative for SARS-CoV-2 [26, 32, 39, 47, 48], with the exceptions of Zhu et al. and Wu et al. who found one positive sample among 5 samples from 5 women [49], and among 3 samples from 3 women, respectively [37]. These preliminary findings suggested that transmission of SARS-CoV-2 through breast milk was unlikely.

Dong et al. reported the presence of IgG and IgA SARS-CoV-2 antibodies in breast milk samples taken from a woman with a positive throat swab test for COVID-19 [50]. This suggested that breast milk could have protective effects against infection with COVID-19, though more evidence is needed for confirmation.

Public health and medical organizations released guidance regarding breastfeeding for mothers with confirmed SARS-CoV-2 infection that weighed infection risk with the known and documented benefits of breastfeeding and early bonding. The WHO and UNICEF recommended continued breastfeeding, rooming in, skin to skin contact, and kangaroo care utilizing infection control practices. Specifically, the “WHO recommends that mothers with suspected or confirmed COVID-19 should be encouraged to initiate or continue to breastfeed. Mothers should be counselled that the benefits of breastfeeding substantially outweigh the potential risks for transmission.” In contrast, the Centers for Disease Control and Prevention, while encouraging the continuation of breastfeeding in general, stated, “temporary separation of the newborn from a mother with confirmed or suspected COVID-19 should be strongly considered to reduce the risk of transmission to the neonate” [51].

### Mental health

Pregnant women and new mothers are more likely to experience mental illness than non-pregnant individuals [52]. Several COVID-19-related studies in India, China, and Italy of the intrapartum and postpartum periods considered clinically relevant anxiety and depression and their symptoms through self-reports and clinical assessments. Additional maternal mental health issues including substance use disorders and hostility aggression have yet to be studied in depth.

The pandemic significantly impacted maternal mental health. Feelings of anxiety and depression were associated with maternal fear of vertical transmission of the virus to their infants, limited accessibility of antenatal care resources, and lack of social support [53, 54]; these experiences also created a source of stress for pregnant and postpartum women without COVID [27, 55]. Social distancing and isolation/quarantine procedures implemented during the pandemic increased risk of psychological problems among pregnant women and new mothers [53–55].

During pregnancy, self-reported rates of clinically relevant anxiety and depressive symptoms were higher among pregnant women relative to their retrospectively self-assessed pre-pandemic levels and when compared to non-pregnant individuals in a multicenter cross-sectional study performed in China by Y. Wu et al. In the same study, thoughts of self-harm were also more frequent than before the pandemic [52]. Additionally, based on a small case series, Kotabagi et al. proposed a positive correlation between both clinically relevant maternal anxiety and depression and the number of COVID-19-related deaths in the population [56]. The unpredictability of COVID-19, along with deprivation of social and family support, increased perinatal distress [57]. A global survey of pregnant and postpartum women by Koenen and colleagues found that 40% of women screened positive for post-traumatic stress disorder (PTSD); over 70% of women also reported clinically significant depression or anxiety [58]. These findings are highly plausible, but must be seen against the background that carefully controlled epidemiological studies are scarce. To establish time trends in psychiatric or trauma prevalence is notoriously difficult as the same population must be assessed with the same measures in the same setting before and during the crises.

The postpartum period was less well-studied than the intrapartum period. Several authors speculated that limited health resources and increased prevalence of home deliveries without trained obstetric clinicians contributed to depression and distress among all pregnant women and new mothers [53, 59]. Jungari reasoned that heightened levels of clinically relevant depression likely arose from maternal fear of infection for both themselves and their infants, social isolation, and uncertainty surrounding viral spread, but empirical evidence was lacking [54].

### Prenatal and postnatal care

The COVID-19 pandemic required postponement of many non “essential” health services to prevent transmission within clinics, which led to significant reductions in the obtention of antenatal and postnatal care. In the US, an online survey of 4451 pregnant women found nearly a third reported elevated levels of stress, with alterations to prenatal appointments cited as a major reason for this elevation. [60]. A modelling study on the indirect effects of the pandemic in 118 LMIC estimated a reduction in antenatal care by at least 18%, and possibly up to 51.9%, and a similar reduction in postnatal care [61].

This estimate was supported by countries’ changes in perinatal care guidelines [62]. A consultant Obstetrician and Gynecologist at the Lagos University Teaching Hospital stated that those in early pregnancy were urged to come in once in eight weeks rather than once in four, and the number of antenatal care visits decreased from 10 to 15 to an average of 6 [63]. Women also chose to forego visits due to lack of transportation, familial pressure to isolate, and personal fears of the virus [64]. Maternal health workers, such as midwives in Kenya, Uganda and Tanzania, reported low numbers attending maternal health clinics, and more women coming into hospitals late, without sufficient antenatal care [65]. A survey by the Population Council sampling heads of households across five Nairobi urban slums found that 9% of participants forewent health services such as antenatal care and immunization/nutrition services for children [66]. Further, a rapid gender analysis by CARE West Africa found consistent reports of false rumors about the virus and a general mistrust of health workers, leading to some men, especially in rural areas, forbidding their wives from seeking health services. In Mali, most female respondents said they were not accessing health services, out of fear of the virus and confusion about which services were still being offered [67].

A global, cross-sectional study of maternal and newborn health professionals by Semaan et al. found a significant reduction in antenatal care services utilized as clinics reduced hours, number of visitors permitted, and in-person visits during pregnancy [68]. In some areas of the UK, women were provided with blood pressure machines and urinalysis sticks to conduct their own antenatal checks. Some antenatal care was offered via telemedicine, however this varied regionally. Respondents from the UK expressed concerns about the impacts of reduced contact on the quality of maternity care, and participants in LMICs recognized women’s inadequate access to communication infrastructure, as telehealth was far more elusive in rural areas, particularly for women [69, 70].

### Healthcare infrastructure

The temporary closure of outpatient clinics during shelter at home orders left many women without access to time-sensitive maternal and reproductive health care, from routine gynecological checkups to prenatal care to abortion. Classifying abortion care as “non-essential” severely restricted access regionally or nationwide in many countries during periods of lockdown [71, 72]. The UN Population Fund estimates that if COVID-19 related disruption continued for 6 months, 47 million women in 114 LMIC will be unable to use modern contraceptives, and an additional 7 million unintended pregnancies will occur globally [73]. Beyond temporary measures, many clinics closed their doors entirely. By April, 5,633 static and mobile clinics and community-based care outlets closed across 64 countries, according to an International Planned Parenthood Federation survey of its national members [74]. Facilities that remained open were overwhelmed, particularly in LMICs, where many hospitals were already overcrowded. Further, pregnant individuals in many LMICs, with particularly dire numbers in India, were turned away from hospitals or denied ambulances and forced to endure labor on the streets or at home [75, 76]. To mitigate this, hospitals limited the number of people per room and the duration of their stay and reduced postpartum stays. However, this mitigation could negatively impact access to and quality of care.

Semaan et al. revealed that many maternal and newborn healthcare providers worldwide did not receive training in COVID-19 from their health facility, and 53% of participants in LMICS and 31% in HICs did not feel knowledgeable in how to care for a COVID-19 maternity patient; 90% of participants reported higher stress levels [68]. This lack of training and confidence hindered quality of care, with the additional burden of staff and supply shortages. Supply chain breakdowns have left many facilities without access to medications or blood products, which are critical to treating postpartum hemorrhage [70, 73, 74].

While some maternal deaths observed during the pandemic were directly caused by COVID-19, a significant portion may have been attributable to underlying factors. Using evidence from a case series of 20 COVID-19-related maternal deaths, Takemoto et al. proposed that inadequacy of the Brazilian healthcare system was responsible for Brazil’s high rate of maternal mortality [77]. In Brazil, antenatal care resources were already limited, and even fewer were available during the pandemic as many were repurposed for the care of COVID-19 patients. Likewise, the system failed to address existing public health issues which increased the risk of maternal mortality resulting from COVID-19 among pregnant individuals. Women and girls in Sub-Saharan Africa were also expected to experience significant secondary consequences from the COVID-19 pandemic, leading to a rise in maternal mortality during the pandemic. [78].

Studies in both Nepal and the United Kingdom of pregnant individuals found the incidences of stillbirth and neonatal mortality were significantly higher during the pandemic period than the pre-pandemic period. Those experiencing stillbirth and infant mortality did not show symptoms of COVID-19, suggesting these outcomes may instead be due to the reallocation of medical resources towards COVID-19 patients and the subsequent reduction in hospitalization for labor management and perinatal care visits [79, 80]. Likewise, another observation is attributed to reduced care: the consistent reductions in preterm birth were seen across various time windows surrounding the implementation of COVID-19 mitigation measures in different countries such as Netherlands, Ireland and Denmark [81] Authors discussed reduced air pollution and maternal stress during pregnancy as potential causal factors; however, a large minority of preterm births, was iatrogenic, suggesting healthcare provider behavior may be a contributing factor. When routine pre-pandemic care was offered, obstetricians may have induced delivery more often due to more close surveillance of pregnancies, usually for maternal or fetal health concerns (e.g. following deviations in cardiotocography). Delivery is more likely to be induced late-preterm, which in the study accounted for all of the prevalence differences [81]. Changes in care-seeking behavior and care availability due to the COVID-19 pandemic may in some contexts lead to potential improved outcomes (reduced preterm delivery), however, this may come with an increase in stillbirth. It is certainly is a major research challenge with potential lessons for obstetric care.

The long-term impacts of the COVID-19 pandemic on maternal health were yet to be determined, but modelling studies indicated potentially grim outcomes particularly for LMICs. Weak healthcare systems in these countries were unable to mount the necessary response to the pandemic, which allowed the virus to spread rapidly [82]. The public health and healthcare sectors in LMICs were chronically under-funded and under-resourced, leaving them ill-prepared to meet the demands of the pandemic and implement the response measures recommended by leading public health organizations [83]. These shortcomings of the healthcare systems in LMICs threatened both the physical and mental health of pregnant and postpartum people.

### Gender equity in the workforce

The social distancing and lockdown measures of the COVID-19 pandemic caused significant consequences for business sectors where interactions between individuals were frequent and often unavoidable. Women were over-represented in these industries; data from the UK Labour Force Survey revealed that approximately 46% and 39% of working women and men, respectively, were employed in critical sectors, while 19% and 13%, respectively, were employed in locked-down sectors [84]. Safety measures to reduce viral spread revealed that many jobs could be carried out remotely; however, certain essential positions required employees to continue to show up in-person and risk exposure. While there was a lack of research on women’s role as essential workers, 2019 research by Boniol et al. found that women comprise 70% of the healthcare workforce worldwide [85]. A New York Times analysis of US census data crossed with the federal government’s essential worker guidelines also found that 1 in 3 jobs held by women were designated as essential [86].

While working an essential job provided job security, it also increased the risk of SARS-CoV-2 transmission, particularly in the healthcare workforce, given the high contact nature of medical care, the higher risk individuals who sought it, and the lack of PPE many hospitals faced [87, 88]. Data show that women tend to bear a larger burden than men of addressing household needs and providing childcare [89]. The burden of domestic work has increased during the Covid-19 pandemic. Approximately nationally representative survey data from Canada and Australia revealed the average Canadian woman with children spent nearly 50 more hours per week on childcare during the pandemic than did the average man, and the average Australian woman with children spent nearly 43 more hours on childcare. Although this disparity in unpaid care work existed prior to the pandemic, childcare needs have increased for many households [90].

In LMICs, the majority of employed women worked in the informal sector, where they did not have access to services such as paid sick leave, maternity leave, or unemployment benefits [61, 91]. Data from UN Women has found that in Kenya, 60 percent of all job losses recorded since the crisis were held by women [92]. Further, a rapid qualitative assessment by Hivos East Africa found that thousands of females, who are often the main household earners in East Africa, have been laid-off due to the pandemic [93], and surveys by Population Council and World Vision International Cambodia found that a higher percentage of women than men have completely lost their income or earning potential [66, 94].

In several but not all HICs, women were still more likely to become unemployed during the COVID-19 crisis. The U.S. Bureau of Labor Statistics reported that in April 2020 alone, women accounted for 55% of the 20.5 million jobs lost in America, and that job loss was more prevalent and occurred at a more rapid rate for women than men [95]. A study by Adams-Prassl et al. on large geographically representative samples of individuals in the US, UK, and Germany found that in the UK and US, women faced a higher likelihood than men of losing their jobs or report lower earnings during the pandemic in comparable jobs, even when controlling for job characteristics such as college degree [96]. Conversely, gender did not serve as a significant predictor of job loss in Germany.

### Domestic violence

Lockdown measures required individuals to stay inside for extended periods of time, and early data demonstrated notable spikes in domestic violence (DV). Police data were consulted as evidence of increased violence, and surges in DV cases were noted in several countries [97–101]. In addition, DV hotlines and charities in many countries also experienced higher influxes of calls since January 2020 [97, 98, 100, 102, 103]. Non-representative surveys from Women’s Safety New South Wales and Foundation Lance d’Afrique Burundi revealed increased requests for help by survivors to female frontline workers [104, 105]. The Chief Justice of Kenya announced that in the first two weeks of April alone, gender-based violence cases increased by over a third [78]. Similarly, data from India’s National Commission for Women shows that domestic violence complaints more than doubled after Prime Minister Modi announced lockdown on March 24, 2020 [92].

There is a lack of representative epidemiological data on increased DV, and the existing data did not specify if the victims were pregnant or mothers. The breadth of reported cases is alarming, and this increase in DV is expected to be detrimental to maternal health outcomes [105–107]. The actual number of DV incidents is likely higher than reported as lockdown measures and fears of virus spread limited community support for women seeking freedom from abusers [101, 108, 109].

## Discussion

This study represents a comprehensive scoping review of the direct and indirect impacts of the COVID-19 pandemic on maternal health. By using broad search terms and inclusion criteria, we were able to review literature on social and economic impacts of the pandemic as well as the physical and mental health impacts. However, this study did not include literature in languages other than English, which likely skews results towards countries in which English is the primary language. It was difficult to locate published information on LMICs pertaining to maternal health. We attempted to mitigate this by including grey literature and news articles from a variety of sources. Finally, while a scoping review methodology allowed us to rapidly synthesize literature, it does not represent as rigorous a process as a systematic review or meta-analysis.

While early results suggested otherwise, recent studies with large sample sizes utilizing control groups suggest that pregnant people experiencing symptoms of COVID-19 are at higher risk of adverse outcomes than those who are not pregnant [17, 18]. Additionally, the non-medical impact of the COVID-19 pandemic is already apparent in this vulnerable population. While short and medium-term consequences of these impacts are emerging, the long-term consequences are currently unknown and will require careful research to be elucidated.

To date, studies of the effects of the COVID-19 pandemic have, perhaps understandably, given time constraints and availability of data, lacked rigorous methods. To adequately assess these effects, we require research that carefully controls for pre-COVID-19 levels of the different outcomes of interest (e.g. depressive symptoms, C-Section) and population characteristics (e.g. comorbidity, socio-economic status) to more validly assess time trends. While reducing the frequency of prenatal visits in high income countries (HIC) may not necessarily be associated with worse birth outcomes, reducing the basic antenatal care in low- and middle-income countries (LMIC) is likely to impact maternal and neonatal health [110, 111]. Continued surveillance and reporting are critical to ascertain whether maternal mortality and morbidity have increased during the pandemic and which populations were affected most severely.

The Covid-19 pandemic has created a multitude of questions regarding the optimal policies to reduce the spread of SARS-Cov-2 while minimizing the unintended detrimental consequences to family wellbeing and gender equity. Salient among these are: in what circumstances should schools and daycares resume care and in what format? Which models of antenatal and delivery care produce optimal outcomes? Which economic relief policies protect gender equity in the workplace and family wellbeing? Heterogeneous and inconsistent application of policies and models for healthcare and childcare delivery both within and across countries, while potentially not ideal for pandemic response, provide a near-natural experiment that helps to explore these questions.

At the same time, policies impacting pregnant and parenting people have been implemented with little evidence. Several of these policies have the potential to significantly harm pregnant individuals’ health and undermine their rights. Most concerning are those that limit emotional support during labor and delivery, mandate early infant separation, and shorten postpartum stays. While the clinical rationale behind high C-section rates among pregnant individuals diagnosed with Covid-19 are unclear, these rates are alarming given that no evidence exists that C-section delivery lowers risk of transmission of SARS-CoV-2 or improves maternal health [81, 112].

These concerns lead us to provide several clear policy recommendations we believe either have sufficient evidence to merit implementation or must be pursued because of ethical and human rights considerations. The first two pertain to healthcare policy. Given the evidence on the paucity of severe outcomes from SARS-CoV-2 infection in newborns, we urge the CDC to align with the WHO’s strong recommendation to keep the mother/infant dyad together even if the mother has a confirmed infection of SARS-CoV-2. Precautions, of course, are warranted, but our opinion is that the overwhelming evidence behind the benefits of early bonding and breastfeeding outweigh the risk of infection in the newborn.

Second, whenever possible, healthcare organizations should consider the mental health impacts of any policies implemented to reduce risk of transmission. Early and convincing evidence currently exists that maternal mental health issues have increased during the pandemic. Policies that limit or eliminate the ability to give birth with a support person present or that are likely to increase distress, potentially exacerbating underlying mental health issues, should be avoided. With many healthcare organizations shortening postpartum hospital stays or providing postpartum visits through telemedicine, there is also the risk that screening for postpartum depression or other mental health issues will be forgotten or glossed over. Healthcare providers should be vigilant of the increased mental health needs of their pregnant and parenting patients.

Finally, daycare and school closures are causing incredible stress and destabilization to caregivers, especially women, who often bear the brunt of childcare duties. These closures along with other workplace related consequences of the pandemic pose a serious threat to gender equity in the workforce. Without serious mitigation through policy, this threat is potentially far-reaching. We strongly recommend that governments prioritize the resumption of schooling under safe conditions and childcare when easing shelter in place or other pandemic-related restrictions. Failure to do so is likely to worsen any short-term losses in women’s employment given women’s disproportionate burden of childcare, and to put vulnerable single-mother and low income households at risk of poverty and food insecurity.

## Conclusion

While rigorous studies have not yet been conducted, early evidence from this scoping review shows that many of the social and economic consequences of the COVID-19 crisis likely affect women more than men. The low risk of mother to child transmission in-utero or via breast milk is well documented. It seems that pregnancy may constitute a particularly vulnerable period for COVID-19, but this requires further confirmation through well designed and implemented research. An increased risk of distress and psychiatric problems during pregnancy and postnatally during the pandemic is likely, but also in this case high-quality evidence is lacking. Likewise, a rise in the prevalence of domestic violence is plausible and supported by several studies, but we need more representative data. Studies of maternal morbidity and mortality are also lacking. Rigorous epidemiological studies must document the health impact of infection with SARS-CoV-2 during pregnancy as well as the changes in health care service and accessibility and their impact on maternal health. This review, however, provides good evidence that mothers with children are more likely to suffer job and income losses during the pandemic than men and women without children. Single mothers in particular are likely to suffer from food insecurity. These socioeconomic consequences for women are similar across many high- and low-income countries.

## Supplementary Information

**Additional file 1.** Pubmed MeSH Terms Utilized for Peer-Reviewed Articles.

## Data Availability

Full review algorithm will be made available.

## Abbreviations

COVID-19: Coronavirus Disease 2019
DV: Domestic violence
LMIC: Low- and middle-income countries
HIC: High income countries
PTSD: Post-traumatic stress disorder
C-section: Cesarean section
SARS-CoV-2: Severe acute respiratory syndrome coronavirus 2
