# Supplementary material for: The impact of the COVID-19 pandemic on maternal and perinatal health: a scoping review
Source: Reprod Health. 2021 Jan 18;18:10. doi: 10.1186/s12978-021-01070-6 (PMC7812564; doi:10.1186/s12978-021-01070-6)
Supplement: Supplementary file 1 — Additional file 1. Pubmed MeSH Terms Utilized for Peer-Reviewed Articles. [file 12978_2021_1070_MOESM1_ESM.docx]

MeSH Terms: ("Pediatrics"[Journal] OR "PLoS One"[jour] OR "Lancet Glob Health"[jour] OR "Environ Health Perspect"[jour] OR "Soc Sci Med"[jour] OR "Public Health Nutr"[jour] OR "JAMA"[jour] OR "Am J Epidemiol"[jour] OR "MMWR Morb Mortal Wkly Rep"[jour] OR "Int J Epidemiol"[jour] OR "Am J Prev Med"[jour] OR "N Engl J Med"[jour] OR "Am J Public Health"[jour] OR "BMJ"[jour] OR "BMJ Open"[jour] OR "Science"[jour] OR "Nature"[jour] OR "Fam Plann Perspect"[jour] OR "BMC Pregnancy Childbirth"[jour] OR "Stud Fam Plann"[jour] OR "Glob Public Health"[jour] OR "Health Policy Plan"[jour] OR "J Glob Health"[jour] OR "Obstet Gynecol"[jour] OR "Am J Obstet Gynecol"[jour] OR "Reprod Health Matters"[jour] OR "Afr J Reprod Health"[jour] OR "BJOG"[jour] OR "MCN Am J Matern Child Nurs"[jour] OR "Birth"[jour] OR "Contraception"[jour] OR "Perspect Sex Reprod Health"[jour] OR "Int Perspect Sex Reprod Health"[jour] OR "Matern Child Health J"[jour] OR "Women Birth"[jour] OR "Safe Mother"[jour] OR "Health Care Women Int"[jour] OR "J Obstet Gynecol Neonatal Nurs"[jour] OR "Arch Womens Ment Health"[jour] OR "Womens Health Issues"[jour] OR "Pract Midwife"[Journal] OR "Midwifery"[Journal] OR "Midwifery Today Int Midwife"[Journal] OR "Midwives"[Journal] OR "Midwives (1995)"[Journal] OR "J Midwifery Womens Health"[Journal] OR "Midwives Chron"[Journal] OR "Paediatr Perinat Epidemiol"[Journal] OR "MCN Am J Matern Child Nurs"[Journal] OR "Obstet Med"[Journal] OR "Seminars in Perinatology"[journal] OR "Human Reproduction Update"[journal] OR "Seminars in Reproductive Medicine"[journal] OR "Clinics in Perinatology"[journal] OR "Journal of Pregnancy"[journal] OR "J Perinatol"[Journal] OR "Matern Child Nutr"[Journal] OR "J Matern Fetal Neonatal Med"[Journal] OR "Matern Health Neonatol Perinatol"[Journal]) AND ((covid [ti] OR coronavirus [ti])) AND ((family planning[ti] OR birth control[ti] OR contraception[ti] OR pregnan*[ti] OR maternal[ti] OR maternal-fetal[ti] OR mother-baby[ti] OR perinatal[ti] OR maternal-child[ti] OR maternity[ti] OR birth center*[ti] OR facility-based delivery[ti] OR skilled birth attendant*[ti] OR childbirth[ti] OR prenatal[ti] OR antenatal[ti] OR postnatal[ti] OR postpartum[ti] OR intrapartum[ti] OR gestational diabetes[ti] OR diabetes in pregnancy[ti] OR breastfeed*[ti] OR "obstetric fistula"[ti] OR labor and delivery[ti] OR Episiotom*[ti] OR c-section[ti] OR cesarean*[ti] OR obstetric*[ti] OR abortion*[ti] OR miscarriage*[ti] OR stillbirth*[ti] OR malaria in pregnancy[ti] OR PMTCT[ti] OR eclampsia[ti] OR preeclampsia[ti] OR labor induction[ti] OR labor augmentation[ti] OR midwifery[ti] OR midwives[ti] OR nurse-midwives[ti]) NOT case report[ti] AND English[lang])
